# The Long-Term Outcome of Boys With Partial Androgen Insensitivity Syndrome and a Mutation in the Androgen Receptor Gene

**DOI:** 10.1210/jc.2016-1372

**Published:** 2016-07-12

**Authors:** A. Lucas-Herald, S. Bertelloni, A. Juul, J. Bryce, J. Jiang, M. Rodie, R. Sinnott, M. Boroujerdi, M. Lindhardt Johansen, O. Hiort, P. M. Holterhus, M. Cools, G. Guaragna-Filho, G. Guerra-Junior, N. Weintrob, S. Hannema, S. Drop, T. Guran, F. Darendeliler, A. Nordenstrom, I. A. Hughes, C. Acerini, R. Tadokoro-Cuccaro, S. F. Ahmed

**Affiliations:** University of Glasgow (A.L.-H., J.B., J.J., M.R., R.S., M.B., S.F.A.), Glasgow G51 4TF, United Kingdom; University Hospital Pisa (S.B.), 56125 Pisa, Italy; Copenhagen University Hospital (A.J., M.L.J.), 2100 Copenhagen, Denmark; University of Luebeck (O.H.), 23562 Luebeck, Germany; Christian-Albrechts-University of Kiel and University Hospital of Schleswig-Holstein (P.M.H.), 24105 Kiel, Germany; University Hospital Ghent and Ghent University (M.C.), B-9000 Ghent, Belgium; State University of Campinas (UNICAMP) (G.G.-F., G.G.-J.), Campinas 13083-970, Brazil; Dana Dwek Children's Hospital (N.W.), Tel Aviv University, Tel Aviv 64239, Israel; Leids Universitair Medisch Centrum (S.H.), 2333 ZA Leiden, The Netherlands; Sophia Children's Hospital (S.H.), Erasmus University Medical Center, 3015 CN Rotterdam, The Netherlands; Marmara University (T.G.), 34722 Istanbul, Turkey; Istanbul University (F.D.), 34452 Istanbul, Turkey; Karolinska Institutet (A.N.), SE-171 77 Stockholm, Sweden; and University of Cambridge (I.A.H., C.A., R.T.-C.), Cambridge CB2 1TN, United Kingdom

## Abstract

**Background:**

In boys with suspected partial androgen insensitivity syndrome (PAIS), systematic evidence that supports the long-term prognostic value of identifying a mutation in the androgen receptor gene (*AR*) is lacking.

**Objective:**

To assess the clinical characteristics and long-term outcomes in young men with suspected PAIS in relation to the results of *AR* analysis.

**Methods:**

Through the International Disorders of Sex Development Registry, clinical information was gathered on young men suspected of having PAIS (n = 52) who presented before the age of 16 years and had genetic analysis of *AR.*

**Results:**

The median ages at presentation and at the time of the study were 1 month (range, 1 day to 16 years) and 22 years (range, 16 to 52 years), respectively. Of the cohort, 29 men (56%) had 20 different *AR* mutations reported. At diagnosis, the median external masculinization scores were 7 and 6 in cases with and without *AR* mutation, respectively (*P* = .9), and median current external masculinization scores were 9 and 10, respectively (*P* = .28). Thirty-five men (67%) required at least one surgical procedure, and those with a mutation were more likely to require multiple surgeries for hypospadias (*P* = .004). All cases with an *AR* mutation had gynecomastia, compared to 9% of those without an *AR* mutation. Of the six men who had a mastectomy, five (83%) had an *AR* mutation.

**Conclusions:**

Boys with genetically confirmed PAIS are likely to have a poorer clinical outcome than those with XY DSD, with normal T synthesis, and without an identifiable *AR* mutation. Routine genetic analysis of *AR* to confirm PAIS informs long-term prognosis and management.

## Abstract (precis)

Using the I-DSD Registry, a study of long-term outcome in young men suspected of having PAIS in childhood reveals that outcome is clearly worse in those with a confirmed AR gene mutation.

Androgens play an important role in a wide range of biological processes including sexual differentiation (1). Defects in androgen synthesis or action in a 46, XY infant can give rise to a very variable phenotype, ranging from normal female external genitalia to various grades of undermasculinization of male genitalia (2). Boys with no evidence of gonadal dysgenesis and normal androgen synthesis have often been considered to have partial androgen insensitivity syndrome (PAIS), a condition that usually arises due to a mutation in the androgen receptor (AR) gene (*AR*) (3). Although PAIS may be the commonest phenotypic entity that is suspected in 46, XY disorders of sex development (DSD), < 30% of boys may have confirmed PAIS with a detectable mutation in *AR* (4). A mutation in *AR* may also be found in boys with relatively minimal signs of undermasculinization (5), and because most of these infants will have no clear evidence of a disorder of androgen synthesis or action (6), it is unclear whether all of these boys merit routine genetic analysis of *AR*. Although there is some evidence that young men with PAIS may have a suboptimal medical and surgical outcome (7), the rarity of this condition has prevented a conclusive study on the long-term clinical outcome of boys presenting with the wide range of phenotype that has been previously reported in boys with a confirmed mutation in *AR* (3). It is possible that androgen insensitivity may be due to a molecular abnormality in a pathway downstream of the AR (8) or in the noncoding region adjacent to *AR* (4). A clear understanding of the long-term phenotype of men with PAIS will also allow improved diagnostic genetic targeting in those cases that have the PAIS phenotype but no detectable mutation in *AR*.

With the advent of the International DSD (I-DSD) Registry (9), a study of the long-term outcomes of rare conditions such as PAIS in a sufficiently large cohort has now become feasible. The current study was performed to assess whether the long-term outcome with a particular focus on genital development and gonadal function in boys with a genetically confirmed *AR* mutation was different from those with a similar phenotype but without an identifiable *AR* mutation.

## Patients and Methods

All 46, XY male patients registered as having PAIS who were under the age of 16 years at the time of diagnosis and > 16 years old at the time of data collection were identified in the I-DSD Registry. The registry is an international database of pseudoanonymized information on people with DSD, and the data are deposited by their clinicians after receiving informed consent from the patients or their guardians. Details of the development of the registry and its recent use have been previously reported (9, 10), and its standard operating protocol is available at http://www.gla.ac.uk/schools/medicine/research/childhealth/i-dsdproject/thei-dsdregistry/standardoperatingprotocol/(accessed March 28, 2016). The registry is approved by the National Research Ethics Service in the United Kingdom as a research database of information that is collected as part of routine clinical care. All I-DSD Registry users who had registered an eligible case were approached for results of *AR* gene analysis, clinical characteristics at first and most recent presentations, biochemical characteristics, and subsequent management. The registry also collects information on the extent of certainty of diagnosis, and this is categorized as “clinical certainty,” “biochemical certainty,” and “genetic certainty.” In this study, all included cases had normal testes function and T synthesis. However, cases of suspected PAIS that did not reach genetic certainty because they did not have a genetic confirmation despite *AR* analysis were not referred to as having PAIS so that they could be differentiated from the cases of PAIS that actually had a mutation in *AR.* Details of the information collected are summarized in Supplemental Table 1. The external masculinization score (EMS) was calculated as previously described (12). Briefly, the EMS is a composite score that is based on the site of the urethral meatus, location of the gonads, the presence of a micropenis, and the presence of labioscrotal fusion; normal male external genitalia as would be expected in a boy would have a score of 12, whereas normal female external genitalia would have a score of 0. Historical records of serum LH, FSH, and T as measured by local immunoassays were collected if available. All cases were studied using full sequencing of the coding regions and intron/exon boundaries (exons 2–8), with some cases undergoing analysis of exon 1.

Continuous variables were described as medians and ranges, and intergroup comparison for these variables was performed by Mann-Whitney *U* tests. A Fisher exact test was performed to compare proportions in different groups. The level of *P* < .05 was considered to be statistically significant, and all analyses were performed using XLStat (Addinsoft).

## Results

### PAIS and AR mutation status

Among the 1892 records in the I-DSD Registry at the time of the study, there were 1225 (65%) cases with a 46, XY karyotype. Of these 46, XY DSD cases, 154 (13%) had been categorized as PAIS, and 60 of these 154 (39%) were identified as men over the age of 16 years. Details on *AR* mutation testing and clinical information were available in 52 of the 60 (87%) men. In this latter cohort, 29 men were identified as having 22 different *AR* mutations within exons 2 to 8 (Table 1) and, therefore, had genetically confirmed PAIS. Five mutations (D565N, Q825K, L712F, R855H, and R846H) were observed more than once, all in related individuals. Despite having the same genetic mutation, the clinical phenotype at initial presentation varied widely (Table 1). One man who presented at birth with distal hypospadias had two *AR* mutations, including one in exon 2 (L545P) and one in exon 5 (T739C). The remaining 23 cases who did not have a mutation in *AR* were, for the purpose of this study, referred to as XY DSD.

**Table 1.** Mutations and Clinical Features of All Genetically Confirmed Cases of PAIS With a Mutation in *AR*

Age at First Presentation	EMS At First Presentation	Clinical Features	Family History	EMS at Last Assessment	Testos Treatment	*AR* Mutation
1 d	2	H, M, B	Y	9	N	R855H
1 d	4	H, M, B	N	4	N	R855H
1 mo	2	H, M, B	N	5	Y	D565N
1 mo	3	H, M, B	Y	6	Y	S598A
1 mo	4	H, M, B	N	6	Y	L839P
1 mo	4	H, M, B	Y	9	N	R855H
1 mo	6	H, B	N	9	Y	G868L*
1 mo	6	H, B	Y	8	Y	L712F
1 mo	6	H, M, U	N	9	Y	L839I*
1 mo	8	H, M, U	N	10	Y	A840C
1 mo	8	M, U	Y	9	Y	L712F
1 mo	10	H	N	12	N	R846H
3 mo	6	H	N	12	N	S597R
3 mo	7	H, M	N	10	Y	L545P*, T739C*
3 mo	8	M, U	Y	9	Y	L712F
1 y	10	H	N	12	Y	A608G
2 y	2	H, M, U	Y	5	Y	D565N
5 y	9	M	Y	12	N	Q825K
6 y	12	None	Y	12	Y	Q825K
11 y	4	H, M, B	N	6	N	F754L
11 y	5	H, G	N	8	Y	Q799E
11 y	12	G	N	12	N	P695S*
12 y	7	H, B	N	9	N	G789A*
12 y	9	G	Y	10	Y	Q825K
12 y	12	G	Y	12	N	R846H
13 y	6	H, M	N	9	N	A596T
13 y	9	M, G	N	12	N	N757S
16 y	9	M, G	N	12	N	Q825K
16 y	12	G	N	12	Y	Q825K

Abbreviations: H, hypospadias; M, micropenis; G, gynecomastia; U, unilateral undescended testis; B, bilateral undescended testes; Testos, testosterone; Y, yes; N, no. All variants are reported in the Androgen Receptor Genes Mutation Database (11) except those marked with asterisks.

### Clinical features at first presentation

**Figure 1.**
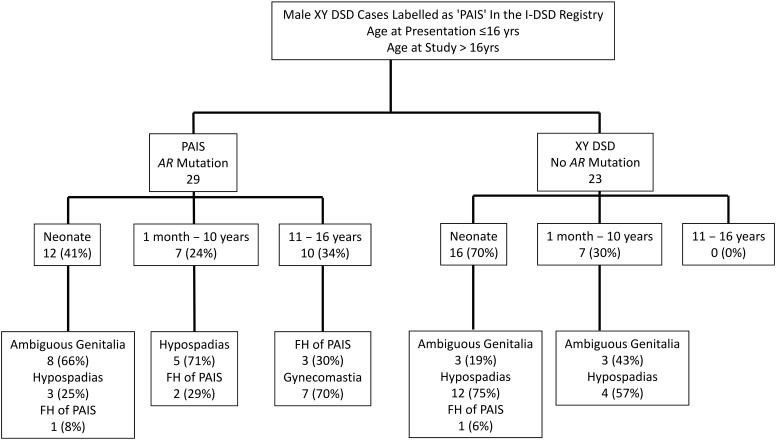
Consort diagram with brief description of main reason for presentation in the genetically confirmed cases of PAIS with a mutation in *AR* (*AR* Mutation) and those cases that were XY DSD with normal androgen synthesis but had no mutation in *AR* (No *AR* Mutation). FH, family history.

Of the 52 men included in this study, the initial presentation was reported as hypospadias in 28 (54%), ambiguous genitalia in 11 (39%), and a positive family history in two (7%). In older boys, gynecomastia was another clinical presentation, but this was only encountered as a reason for presentation in those with an *AR* mutation (Figure 1); none of the XY DSD cases without an *AR* mutation presented in adolescence, and because of this skew, the median age at presentation tended to be higher in the group with an *AR* mutation (Table 2). There was no significant difference in the number of cases with or without an *AR* mutation who presented with hypospadias, undescended testes, or micropenis, and the median EMS at first presentation in the two groups was similar at 7 (2, 12) and 6 (2, 12), respectively. On excluding the cases who had presented with a positive family history or gynecomastia, the median EMS in the group with an *AR* mutation and those without was similar at 5 (2, 10) and 6 (2, 12), respectively.

**Table 2.** Comparison of Clinical Characteristics of Genetically Confirmed Cases of PAIS With a Mutation in *AR* and Cases That Were XY DSD With Normal Androgen Synthesis But Had No Mutation in *AR* at First Presentation and Last Assessment

	*AR* Mutation	No *AR* Mutation	*P*
n	29	23	
Age at first presentation, y^a^	0.3 (0, 16.4)	0.1 (0, 10.0)	.05
Age at last assessment, y^a^	21 (16, 52)	24 (18, 30)	.64
EMS at first presentation^a^	7 (2, 12)	6 (2, 12)	.9
EMS at last assessment^a^	9 (3, 12)	10 (7, 12)	.28
Hypospadias at first presentation (Prox, Mid, Dis, NK)	20 (69) (15, 2, 1, 2)	20 (87) (13, 0, 6, 1)	.19
Hypospadias at last assessment	7 (24)	3 (13)	.12
Undescended testis at first presentation	2 (7)	0 (0)	.49
Undescended testis at last assessment	2 (7)	0 (0)	.49
Bilat undescended testes at first presentation	7 (24)	11 (48)	.09
Bilat undescended testes at last assessment	0 (0)	0 (0)	1
Micropenis at first presentation	13 (45)	6 (26)	.25
Micropenis at last assessment	5 (17)	1 (4)	.21
Gynecomastia at first presentation	7 (24)	0 (0)	.01
Gynecomastia at last assessment	29 (100)	2 (9)	<.001

Abbreviations: Bilat, bilateral; Prox, proximal; Mid, midshaft; Dis, distal; NK, not known. Data are expressed as number (percentage) unless specified otherwise.

a Continuous variables expressed as median (range).

### Clinical biochemistry at first presentation

Serum T tended to be higher in subjects that had an *AR* mutation, but the difference was not statistically significant (Table 3). Of 29 cases with an *AR* mutation, serum LH and FSH were available in 23 and 22 cases, respectively, and there were also no significant differences between the cases with and without an *AR* mutation. Although the median LH:FSH ratio in those with and without an *AR* mutation was similar at 1.1 (0.01, 102) and 1.3 (0.3, 6.7), respectively, the T:LH was significantly higher at 1.9 (0.1, 13.3) compared to 0.9 (0.1, 1.7) in those with a mutation (*P* = .02) (Table 3). In a separate comparison of the 15 infants who presented before the age of 6 months and who had an *AR* mutation and the 16 infants without an *AR* mutation, none of the biochemical values above showed any statistical difference.

**Table 3.** Comparison of Biochemical Characteristics of Genetically Confirmed Cases of PAIS With a Mutation in *AR* and Cases That Were XYD DSD with Normal Androgen Synthesis But Had No Mutation in *AR* at First Presentation and Last Assessment

	*AR* Mutation	No *AR* Mutation	*P*
Age at first presentation, y	0.3 (0, 16.4)	0.1 (0, 10.0)	.05
Age at last assessment, y	21 (16, 52)	24 (18, 30)	.64
Serum LH at first presentation, IU/L	4.5 (0.04, 21.1) (23)	3.3 (0.1, 6.7) (9)	.32
Serum LH at last assessment, IU/L	11.2 (1.8, 57) (24)	4.3 (0.1, 7.7) (9)	.002
Serum FSH at first presentation, IU/L	1.9 (0.1, 39.8) (22)	1.7 (0.2, 5.5) (11)	.5
Serum FSH at last assessment, IU/L	4.7 (1.2, 89.0) (22)	5.6 (0.3, 12.8) (7)	.56
Serum LH:FSH at first presentation	1.1 (0.01, 102) (16)	1.3 (0.3, 6.7) (5)	.77
Serum LH:FSH at last assessment	1.7 (0.2, 13.5) (18)	0.7 (0.1, 2.3) (7)	.22
Serum T at first presentation, nmol/L	8.6 (0.01, 60.8) (23)	2.8 (0.1, 21.5) (15)	.09
Serum T at last assessment, nmol/L	18.7 (4.8, 68.3) (24)	10.2 (3.6, 23.4) (8)	.03
Serum T:LH at first presentation	1.9 (0.1,13.3) (21)	0.9 (0.1, 1.7) (7)	.02
Serum T:LH at last assessment	2.3 (0.2, 12.7) (24)	1.8 (0.8, 3.2) (4)	.57

Results of these parameters were available in a variable number of cases and are expressed as median (range) (number).

### Current clinical status

**Figure 2.**
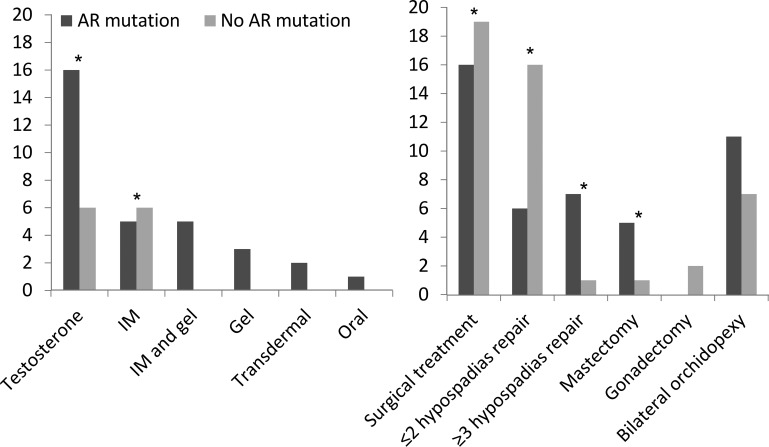
Testosterone therapy (left) and surgical encounters (right) in genetically confirmed cases of PAIS with a mutation in *AR* (*AR* mutation) and those cases that were XY DSD with normal androgen synthesis but had no mutation in *AR* (No *AR* mutation). *, *P* < .05.

Irrespective of *AR* mutation status, undermasculinization was frequently observed at the most recent clinical presentation (Table 2). On excluding the men who had presented because of a positive family history or gynecomastia, the median EMS values in the cases with and without an *AR* mutation were 6 (3, 12) and 9 (2, 12), respectively. In the whole cohort of 52 cases, 25 (42%) had required T therapy at puberty, and the regimen that was used was very variable (Figure 2). Testosterone administration was more common in the men with an *AR* mutation (Figure 2). Of the 16 men with an *AR* mutation and six without an *AR* mutation who had T therapy, a micropenis in adulthood was reported in two (13%) and zero (0%) cases, respectively. Of the 29 cases who had an *AR* mutation, 20 had hypospadias at initial presentation and six (33%) continued to have a hypospadias despite surgical repair, whereas only one of the 20 with hypospadias and no *AR* mutation continued to have a hypospadias (Table 2). In addition, those with an *AR* mutation were more likely to have a greater number of surgical procedures for their hypospadias repair (Figure 2), ranging up to 10 in one man. All men with an *AR* mutation had gynecomastia, compared to only 10% of men without a mutation (Table 2). Six men had gynecomastia that was severe enough to require mastectomy (Figure 2); five (83%) of these six men had an *AR* mutation. Among the cases without a mutation, there was one person who had micropenis, one who had persistent hypospadias, and two with gynecomastia (Table 2).

### Clinical biochemistry at last assessment

In those with an *AR* mutation, serum LH and T were significantly higher than in those without an *AR* mutation (Table 3). Both LH:FSH and T:LH ratios tended to be higher in men with a mutation, and the difference reached statistical significance for the latter (Table 3). Using LH and FSH values of 12 IU/L as a reference for 2 SD above the mean for healthy men (13), it was apparent that in those with an *AR* mutation, serum LH and FSH were higher than this cutoff in 10 of 24 (42%) and seven of 22 (32%) cases, respectively. Only one of these 18 men had a history of bilateral undescended testes. Of the men with no *AR* mutation, none of nine had a serum LH above 12 IU/L, and two of seven (29%) had a raised FSH. Both LH:FSH and T:LH ratios tended to be higher in men with a mutation, but the difference was not significantly different (Table 3).

### Other outcomes

The provision of psychological input was reported in 11 of 52 (21%) cases, and seven of these 11 men had an *AR* mutation. Of these 11 men, one (9%) had declined a psychological referral, eight (73%) were receiving psychological support at the last assessment, and two (18%) had previously received input but were no longer actively involved with this. Fertility data were available for 14 men with an *AR* mutation and four without a mutation who were between 18 and 34 years of age at the time of the most recent presentation. Among this cohort, two with an *AR* mutation had undergone sperm analysis that had demonstrated azoospermia (FSH, 89 and 86 IU/L at last presentation). One of these men had one undescended testis requiring orchiopexy, and the other man had undergone bilateral orchiopexy. Only two men had offspring; one of these men did not have an *AR* mutation and had required assisted conception after sperm analysis demonstrated oligospermia, and the other, who had an *AR* mutation, had conceived naturally. None of the men in this study had any record of tumor-related events. Two of the 29 men with an *AR* mutation (7%) were noted to have obesity with body mass indices of 28 and 30 kg/m^2^; one had undergone assessment for possible bariatric surgery, and the other had refused any dietetic advice. None of the men had any recorded investigations for concerns regarding bone mineral density or cardiometabolic health.

## Discussion

The current report represents the largest study on clinical outcomes in young men with XY DSD and normal T synthesis and who were previously suspected of having PAIS in childhood and adolescence. The study not only raises concerns about the long-term outcome in those men with PAIS but also strengthens the clinical rationale for the routine genetic analysis of *AR* in boys where there is a clinical suspicion of PAIS.

Access to the I-DSD Registry allowed the identification of a sufficiently large group of male cases of 46, XY DSD with a variable extent of undermasculinization at initial presentation and who had a clinical phenotype consistent with PAIS. The difficulty in diagnosis was illustrated by the fact that there was no clear difference in the physical and biochemical features that were assessed at presentation in those cases with an identifiable mutation in *AR* compared to the ones without a mutation. Although higher T concentrations and T:LH ratios were encountered in the cases with an *AR* mutation, there was a large overlap between the two groups and as observed previously (13, 14).

As young men, those with an *AR* mutation tended to have a worse prognosis for virilization as reflected by the gynecomastia. These men were also more likely to have hypospadias and micropenis, but this difference did not reach statistical significance. In addition, these men with an *AR* mutation were more likely to have had T therapy. A variable level of virilization after androgen therapy has been reported in PAIS (8) and may reflect the lack of standardization of therapy, with reports often limited to single case reports (16). This variability in virilization in PAIS and the wide range of androgen regimens that are used were clearly evident in the current study. The gynecomastia, which was universally encountered in all the young men with an *AR* mutation, confirms previous reports (8, 17). Given that these men have severe and persistent gynecomastia that is more likely to lead to mastectomy, early knowledge of *AR* genetic status would allow the provision of informed guidance to the adolescent.

The current report has identified a clear association between *AR* status and the outcome of hypospadias surgery. Most cases of hypospadias require one or two surgical procedures (18); however, the current study clearly shows that the young men with an *AR* mutation were more likely to have multiple procedures. Given that the relative proportions of the type of hypospadias was similar in the two study groups and that the cases were spread over all the centers, the different outcome cannot be due to case or operator bias. Thus, the poor results are more likely to be related to defective androgen signaling and its links to tissue healing (19). Large-scale studies of hypospadias outcome regularly report a measurable incidence of failed repair (18, 20), but the genetic status of these cases has not been reported and deserves further study. It is possible that the knowledge of the status of *AR* may guide the clinician toward a different approach to surgical management and may also allow improved preparation and counseling of the patient. One-fifth of the studied cohort was reported to have been offered clinical psychology input and, given the concerns raised previously about psychosexual outcome (21) and gender dysphoria (22) in adults with PAIS, we would recommend that clinical psychology assessment and input should be routinely offered, especially in those cases that have a mutation in *AR.*

Previous cross-sectional studies of the hypothalamic-pituitary-gonadal axis in genetically confirmed cases of PAIS, performed at a range of ages in childhood and adolescence, have reported concerns about testicular function, as reflected by a blunted T rise after human chorionic gonadotropin stimulation as well as raised gonadotropins (5, 7). The description of raised gonadotropins has primarily been highlighted for LH and was attributed to a lack of androgen feedback on LH secretion (15, 23, 24). A raised LH and T level may be a clue to the diagnosis of androgen insensitivity syndrome, and although the absolute values of these markers were not remarkable in the current study, the T:LH ratio was higher at first presentation in the cases with an *AR* mutation. It is likely that suppression of LH secretion is partially dependent on both T and estradiol (25), and the raised T:LH ratio may be a reflection of androgen insensitivity in a situation where T can still be aromatized to estradiol. Although the current study shows that LH is more likely to be raised at last presentation, in a substantial subset of cases, FSH was also raised, highlighting the possibility of progressive primary gonadal failure in boys with PAIS. Although in some cases the testicular failure may have been related to maldescent of the testis or the subsequent orchiopexy, most of the cases with high gonadotropins did not have undescended testes, and it is possible that this biochemical picture may reflect the role of androgens in testes development and maintenance (26, 27). This is in line with the histological observation that testes in men with PAIS are prone to fibrosis and loss of testicular architecture (our unpublished observation).

Although over 400 *AR* mutations have now been described in androgen insensitivity syndrome (11), the correlation between phenotype and genotype has remained very poor, with even wide phenotypic variability between individuals with the same mutation and within the same family (28); this variability was also observed in the current cohort. It is still possible that knowledge of a specific *AR* mutation as well as the function of the defective AR protein may assist management (29). However, the current data suggest that the identification of an *AR* mutation in cases of XY DSD may itself be sufficient to predict poor prognosis and personalize long-term management. A recent survey of pediatric endocrine centers has revealed that 43% would routinely consider *AR* analysis in 46, XY infants with genital ambiguity (30). Given that the cases in the current study had a variable level of undermasculinization, our experience would suggest that the threshold for considering genetic analysis for *AR* should be lowered in all cases of hypospadias that are proximal or associated with micropenis or undescended testes, especially when there are no associated concerns about T synthesis. In terms of EMS, this would be equivalent to a score of 11 or below and consistent with other recommendations (31). Other features, such as a positive family history or the presence of gynecomastia in an adolescent, would also lower the threshold.

Knowledge of the biology and genetics of sex development as well as the technology employed for diagnostic genetics has progressed considerably since the period when the cases in this cohort were analyzed (32). Almost all of the cases in the current cohort would have been analyzed in research laboratories with different protocols and different levels of success at sequencing the first exon of *AR* and with variable depth of coverage of the intronic region. The current study emphasizes the need for revisiting the genetic analysis of *AR* in those adolescents and young men who did not have a mutation identified before but who may have features such as gynecomastia, persistent hypogonadism, and poor outcome after hypospadias repair. With the availability of quality accredited laboratories offering analysis of a wide panel of genes associated with sex development as well as increased reliance on whole-genome sequencing (33), it is quite likely that further defects in *AR* or mutations in other genes associated with DSD may be revealed. By targeting analysis in young men with a clearer phenotype of androgen insensitivity, it is also possible that future research can be more effectively directed at understanding the wider clinical relevance of the androgen-signaling pathway downstream of *AR* as well as the noncanonical pathways of androgen signaling (34).

In older men with hypogonadism, cardiovascular morbidity, impaired glucose tolerance, and osteoporosis pose the greatest health and economic burden (35), and there is sufficient evidence that young men with primary hypogonadism may also suffer from these effects (36–39). Even mildly impaired Leydig cell function in otherwise healthy subjects with low-normal T and elevated LH levels is associated with increased mortality and morbidity (39). Given that the men with confirmed PAIS with a defect in *AR* may be severely hypogonadal, as suggested by the current findings, the need for careful monitoring of the wider cardiometabolic consequences of hypogonadism requires strong consideration. Successful fertility has been reported in men with PAIS using assisted conception techniques (17, 40). In the current study, one of the young men with an *AR* mutation was reported to have an offspring naturally, indicating that spontaneous fertility is possible in young men with confirmed PAIS.

Although there are inherent weaknesses in performing a retrospective study that relies on data collected from multiple centers, the power of the study rests in the size of the cohort and the small number of clear and measurable outcomes that were collected. Although it is reassuring that all the participating centers routinely collected the information that was sought, the current study highlights the need for a standardized assessment of all boys who are suspected of having long-term hypogonadism, especially as they approach the age of puberty. It is possible that the cases entered into the registry suffered from selection bias and inclusion of the cases that were older at presentation also introduced further bias. However, this risk was mitigated by the discrete comparison of the group of children who presented at an early age. Increasingly, the diagnosis of PAIS is only reserved for those cases that have a confirmed mutation in *AR*, and it is possible that a number of cases with no *AR* mutation have not been entered in the I-DSD Registry because PAIS and differences in the use of the registry may have introduced further bias. However, it is likely that this change in diagnostic practice would apply to younger cases than those reported here. Finally, a genotype-phenotype correlation was not explored in this study, but this will become more feasible as the number of cases with genetically confirmed PAIS that reach adulthood increases over the next decade.

In conclusion, this study represents the largest cohort to date of young men who presented in childhood with clinical features suggestive of PAIS. There is clear evidence that genetically confirmed cases of PAIS with a mutation in *AR* are more likely to have a worse medical and surgical outcome as young men. Thus, routine genetic analysis of *AR* in boys suspected of PAIS is recommended to guide long-term prognosis and tailor management.
